# Utilization of isoniazid prophylaxis therapy and its associated factors among HIV positive clients taking antiretroviral therapy at Fre Semaetat primary hospital, Hawzien districts, Tigrai, Northern Ethiopia

**DOI:** 10.1186/s40794-020-00106-2

**Published:** 2020-06-17

**Authors:** Haftom Legese, Hagos Degefa, Aderajew Gebrewahd, Haftay Gebremedhin

**Affiliations:** 1 Department of Medical Laboratory Sciences, College of Medicine and Health Sciences, Adigrat University, Adigrat, Ethiopia; 2 Department of Public Health, College of Medicine and Health Sciences, Adigrat University, Adigrat, Ethiopia

**Keywords:** Isoniazid prophylaxis, HIV positive, Antiretroviral therapy, Ethiopia

## Abstract

**Background:**

Isoniazid prophylaxis therapy is a significant public health intervention to prevent the progression of latent tuberculosis to active tuberculosis disease among people living with HIV. Those with HIV are at high risk to develop active Tuberculosis from latent Tuberculosis than those without HIV. Even though there is strong evidence supporting Isoniazid Prophylaxis therapy for Tuberculosis prevention, there is limited information about the implementation of isoniazid prophylaxis therapy in Ethiopia as well as in the study area.

**Objective:**

To determine the effects of Isoniazid Prophylaxis therapy and its associated factors among HIV positive clients taking antiretroviral therapy at Fre Semaetat primary Hospital, Hawzien districts, Tigray, northern Ethiopia.

**Method:**

Institutional based cross-sectional study design was conducted from April to August 2019 among HIV positive clients who came to Fre Semaetat primary Hospital. Data related to socio-demographic characteristics and associated risk factors were taken from 372 HIV positive clients who were selected by a simple random sampling method. Data was coded and cleaned by using SPSS version 23.0 for the final analysis.

**Results:**

A total of 372 HIV positive clients taking antiretroviral therapy were included in the study. Of those, the overall prevalence that took and completed their Isoniazid Prophylaxis therapy for 6 months was found to be 231(62.1%). From those who completed Isoniazid Prophylaxis therapy (IPT), 13(3.5%) was developed active Tuberculosis (TB) incidence. Gender, co-trimexazol Prophylaxis therapy users, HIV positive clients who took Anti-pain and married clients were the predictor among statistically significant variables of Isoniazid Prophylaxis therapy.

**Conclusions:**

Isoniazid Prophylaxis therapy utilization found to below. Therefore, health education and counseling of patients who are in their first 2 months of therapy should be strengthened further. Prophylaxis should be given by service providers, medication side effects should be addressed rapidly.

## Background

Tuberculosis (TB) remains a major public health concern particularly in those countries with poor access to resources [1, 2]. It is the ninth leading cause of death worldwide and the leading cause of a single infectious agent, ranking above HIV/AIDS [3]. The risk for TB is 20–37 times greater among persons infected with HIV comparing to those who do not have HIV infection [4].

The proportion of TB and HIV co-infection is highest in African countries. Overall, the African region accounted for 82% of TB cases among HIV positive individuals in some countries in sub-Saharan Africa, up to 1.68 million killed by TB infection, 0.38 million death were HIV positive [5]. Ethiopia is among the high TB/HIV burden countries with estimated incidence and prevalence of TB 207 and 200 per 100,000 populations as co-infection among HIV positive clients respectively [6].

TB transmission and control measure has faced challenges due to co-infection with HIV. HIV and TB service delivery is insufficiently integrated, and too many people are losing their lives because they are unable to either prevent TB or access life-saving medications for both diseases [7, 8]. Therefore, establishing and strengthening of coordination mechanisms for the providing of ART and Isoniazid Prophylaxis therapy (IPT) to people living with HIV who do not have active TB is essential in order to prevent the transmission and develop of active TB infection [9, 10].

Despite substantial evidence about the effect of IPT or IPT-ART combination therapy in HIV patients, the global utilization rate is far lower than would be expected. Provision of isoniazid preventive therapy (IPT) is one of the public health interventions for the prevention of TB in HIV infected individuals. Even though, According to 2014 WHO report that only 41% of eligible HIV patients used IPT which is very low coverage [11].

However, in Ethiopia, the coverage and implementation of IPT are limited as previous research is done [12], and there is no published data in the study area. Therefore, this current study would be important to determine the effectiveness of the provision of IPT for HIV infected individuals in preventing occurrence of TB in the study area.

## Methods

### Study design, period and area

The institutional-based cross-sectional study design was conducted from April to August 30, 2019. This study was conducted in Fre Semaetat primary hospital, Tigrai, North Ethiopia. Which is located 867 km far from Addis Ababa capital city of Ethiopia to north direction and 107 km to east direction from Mekelle which is the capital city of Tigrai region. Based on the 2007 national census conducted by the central statistical agency of Ethiopia (CSA), this district had a total population of 117,954, of whom 47.8% men and 52.2% were women.

#### Eligibility criteria

HIV positive clients who did not develop active TB based on the TB screening criteria were considered as inclusion criteria.

#### Exclusion criteria

In a client who had incomplete data records (patients who lost during follow up, absence of client’s medical, clinical and laboratory values). Clients who were transferred out from the study area to other health facilities were excluded from the study. Clients already developed TB/HIV co-infection from the documentation.

### Sample size determination and sampling procedure

The sample size was determined using the single population proportion formula.

$$ \mathrm{n}=\frac{{\mathrm{Z}}^2\upalpha /2\ \mathrm{P}\ \left(1\hbox{-} \mathrm{P}\right)}{{\mathrm{d}}^2} $$

By the prevalence of IPT from the previous study done in Arbaminch hospital, South Ethiopia (68%) which was done by Ashenafi A, [13]. Then with a margin of error (5%), (d = 0.05) and 95% level of confidence (z = 1.96).

The total 372 was selected Using a simple random sampling technique to select study participants.

### Study variables

#### Dependent variables

Utilization of IPT.

#### Independent variables

Client related factors, Socio-demographic variables including (Age, Sex, Religion, educational status, marital status, and occupational status) adherence of IPT, social drug use, Drug factors, ART, CPT, anti-pain medication, anthelmintic, and antifungal.

Disease factors, OIs, WHO clinical stage, CD4 count, BMI, and viral load.

### Data collection instruments

Quantitative data was collected through interviewer-administered Face to face technique and were collected from HIV care/ART follow-up clients in ART clinic as well as laboratory data using a structured questionnaire such as socio-demographic characteristics (Age, Sex, Religion, educational status, marital status, and occupational status), medication-related factors (social drug use, Drug factors, ART, CPT, anti-pain medication, anthelmintic, and antifungal) and included based on IPT status, and patients who had a registered baseline such as disease factors (OIs, WHO clinical stage, CD4 count, BMI, and viral load). The questionnaire was adopted from Ethiopia and Tanzania published articles [13, 14].

### Data quality control

To attain the data quality control issue training was given for data collectors and supervisors using the local language. Questionnaires originally developed in English then translated to local language (Tigrigna) and back-translated to English by a different translator who was blinded to the original questionnaire to check for its consistency. Three data collectors were selected from a different facility qualified in bachelor of public health officer and experience of a minimum of 3 years in the HIV clinic. The training was given using lecture and demonstration on data collection procedure and supervision was given for 2 days. The questionnaire was pre-tested 5% of the total sample size at different facilities involving enumerators and supervisors and data was collected at the study hospital.

The principal investigator and supervisors on a daily basis checked and visit to review questionnaires’ completeness, accuracy and consistency and corrective discussion was undertaken with research team members on gaps identified. A reminding remark was given during each morning by the supervisor on how to minimize errors and timely corrective actions were taken. Two data clerks did double data entry and consistency of the entered data was crosschecked by comparing the two separately entered data on Epi-Data version 3.02.

### Data processing and analysis

After the data collected and checked for its completeness, it was coded, entered and cleanedusing EPI-data software version 3.1 then exported to SPSS software version 23 for analysis. Descriptive statistics like frequency, percentage, measures of central tendency and measures of dispersion were carried out. Missing values were analyzed by using multiple imputation techniques. Then the information was presented using frequencies tables and figures. Chi-square test (X^2^) and Bi-variable logistic regression analysis were done for each variable and variables with their *p*-value < 0.025 were taken to the multivariable analysis to control all possible confounders. The multi co-linearity test was carried out to see the correlation between independent variables using standard error. In the multivariable logistic regression analysis, the model fitness test was checked using Hosmer and Lemeshow test. Odds ratios along with 95% CI were used to measure the association between the dependent and independent variables and the level of statistically significant was declared at *p*-value *<* 0.05.

### Ethical consideration

Ethical approval was obtained from the Institutional Health Research Ethics Review Committee (IHREC) of, Adigrat University College of Health and Medical Sciences. A written officiala letter was taken from IHREC to Hawzen district Health Bureau to deem its legality.After getting permission a written official letter was also taken to director of the Fre Semaetat primary Hospital, Hawzen administrative offices and then to the ART clinic officer for permission. The confidentiality of information was secured and no personal identifier was included in the data collection tool.

## Results

### Socio-demographic data

A total of 372 study subjects were included in the study. The majority of 200 (53.80%) respondents were male. Of the total study participants, 252 (67.70%) were aged above 34 years. The mean age of the participants was 37.94 ± 12.15, and the median age was 39. Most of the 306 (82.30%) of the study participants were Orthodox religions followed by Muslim 66 (17.70%). Two hundred twenty-three (59.90%) study participants were unable to read and write. While 149 (40.10%) participants have educated college and above. Among the study participants, 284 (76.30%) were married (Table 1).

**Table 1:** Socio-demographic characteristics of the study participants in Fre Semaetat primary Hospital Hawzen, Tigrai, Ethiopia from April to August 2019 (*N* = 372)

Socio-demographic characteristic’s		Frequency	Percent
**Sex**	Male	200	53.80
	Female	172	46.20
**Age**	0–14	16	4.30
	15–24	35	9.40
	25–34	69	18.50
	> = 35	252	67.70
**Religion**	Orthodox	306	82.30
	Muslim	66	17.70
	Protestants	0	0
**Marital status**	Married	284	76.30
	Single	53	14.20
	Divorced	25	6.70
	Widowed	10	2.70
**Educational level**	Cannot read and write	223	59.90
	Primary	109	29.30
	Secondary	38	10.20
	College and above	2	0.50

### Clinical, immunological and laboratory values

Chi-square test (X^2^) showed that WHO clinical stage (*p* < 0.002), and CD4 count (*p* < 0.01), Hgb(*p* < 0.009), weight (*p* < 0.001) and adherence to ART (*p* < 0.02) were statistically significant with IPT utilization (Table 2).

**Table 2:** Baseline clinical characteristics of clients on IPT among HIV positive clients in Fre Semaetat primary Hospital Hawzen, Tigray, Ethiopia from April to August 2019 (*N* = 372)

Baseline clinical characteristics		IPT take		Total	*P*-value
		Yes	No		
**WHO C/S**	One	238(74.1)	42(82.40)	280	0.002*
	Two	67(20.9%)	9(17.6%)	76	
	Three	3(0.9)	0(0.0%)	3	
	Four	13(4.0)	0(0.0%	13	
**CD4 Count**	< 350	94(29.3%)	14(27.5%)	108	0.010*
	350–499	156(48.6%)	27(152.9%)	183	
	> = 500	71(22.1%)	10(19.6%)	81	
**BMI**	< 18	91(28.3%)	8(15.7%)	99	0.094
	18–25	216(67.3%)	42(82.4%)	258	
	> = 25	14(4.4%)	1(2.0%)	15	
**Hgb**	< 12 mg/dl	39(12.%)	0(0.0)	39	0.009*
	> = 12 mg/dl	282(87.9%)	51(100.0%)	333	
**Weight**	< 40 kg	34(10.6%	0(0.0)	34	0.000*
	40–49	209(65.9%)	29(56.9%)	238	
	50–59	56(17.4%)	21(41.2%)	77	
	> 60	22(6.9%)	1(2.0%)	23	
**Adherence to ART med.**	Good	200(62.3%)	34(66.7%)	234	0.020*
	Fair	80(24.9%	17(33.3%)	97	
	Poor	41(12.8%)	0(0.0)	41	

### Drug-related information

**Fig. 1:**
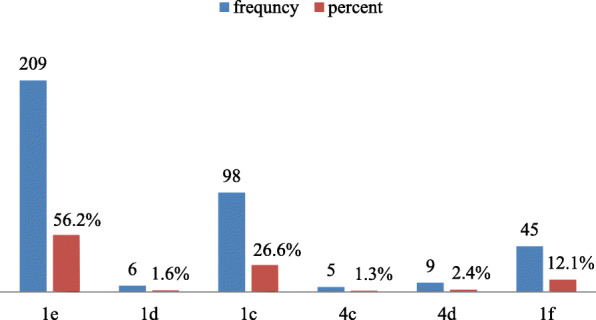
Type of ART medication used among HIV positive clients in Fre Semaetat primary Hospital Hospital Hawzen,Tigray, Ethiopia, 2019

Majority 291(78.20%) of the respondents were received their CPT and 290 (78.00%) study participants were also used anti-pain. All of the participants were taking ART medications, of which 187(85%) were on TDF/3TC/EFV based regimen (Fig. 1).

### Opportunistic infections (OIs)

**Fig. 2:**
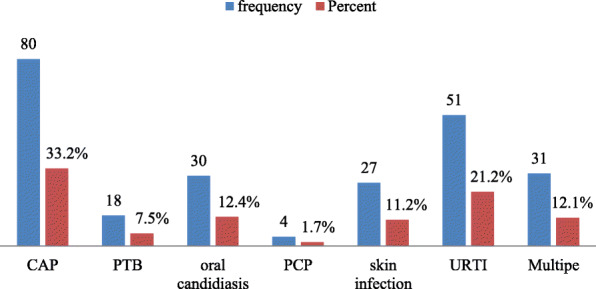
Opportunistic infections among HIV positive clients in Fre Semaetat primary Hospital Hospital Hawzen,Tigray, Ethiopia, 2019

From the total respondents, 241(64.80%) of clients developed opportunistic infections after they taking ART medication. Among the total OIs, 80(33.20%), were community-acquired pneumonia and 51(21.16%) were URTI (Fig. 2).

### Completion rate of IPT for 6 months duration

**Fig. 3:**
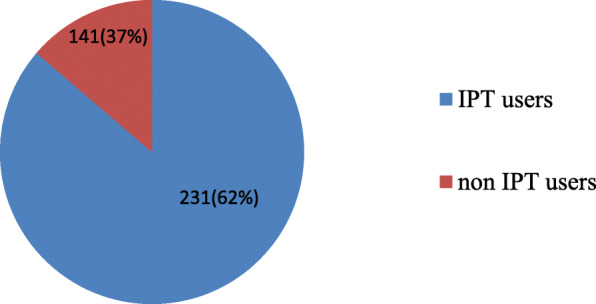
IPT utilization among HIV positive clients taking ART medication in Fre Semaetat primary Hospital Hawzien,Tigray, Ethiopia, 2019

The proportion of HIV positive clients who took and completed for 6 months of their IPT prophylaxis was 231(62%), among those 13(3.5%) participants developed TB. Of the 13 TB positive individuals, 11(84.6%) were developed TB before completing their IPT and 2 (15.40%) were developed TB after completing their IPT (Fig. 3).

### Factors associated with IPT

Bivariate and multivariate logistic regression analyses were performed to assess the association between dependent and independent study variables. According to the bivariate analysis: being female, social drug use, age, marital status, CPT, anti-pain, and antifungal were showed association with IPT and transported to multivariate analysis. Multivariate logistic regression was done to assess the association between the socio-demographic variable, medication and IPT utilization. Sex, anti-pain, marital status, and CPT had statistically significant and were independently associated with IPT utilization. Being males (AOR = 0.034, 95%CI: 0.015–0.077). Use of (AOR = 8.693, 95%CI: 3.600–20.991).HIV positive clients who took Anti-pain (AOR = 3.59, 95% CI: 1.162–11.109). Being Married (AOR = 0.335, 95%CI: 0.140–0.806)] was significantly associated with IPT (Table 3).

**Table 3:** Bivariate and multivariable analysis for IPT utilization among HIV positive clients who use ART medication in Fre Semaetat primary Hospital Hawzien, Tigray, Ethiopia from April to August 2019 (*N* = 372)

Variables	Category	IPT utilization		COR(95% CI)	*P*-value	AOR(95%CI)	*P*-value
		No	Yes				
**Sex**	Male	40	159	3.705(1.836,7.478)	0.000	0.034(0.015,0.077)	0.000*
	Female	11	162	1		1	
**Age**	0–14	3	13	2.402(0.636,9.077)	0.196	0.967(0.147,6.364)	0.972
	15–24	12	23	5.431(2.383,12.377)	0.000	2.998(0.676,13.298)	0.149
	25–34	14	55	2.650(1.274,5.508)	0.009	2.242(0.950,5.291)	0.065
	> = 35	22	230	1		1	
**Marital status**	Married	25	259	0.456(0.267,0.780)	0.004	0.335(0.140,0.806)	0.016*
	Single	26	62	1		1	
**Social drug use**	Yes	14	35	1		1	
	No	37	286	3.092(1.523,6.277)	0.002	0.541(0.214,1.369)	0.195
**Cpt**	Yes	14	277	0.060(0.030,0.120)	0.000	8.693(3.600,20.99)	0.000*
	No	37	44	1		1	
**Analgesics**	Yes	47	243	3.772(1.317,10.80)	0.130	3.592(1.162,11.109)	0.026*
	No	4	78	1		1	
**Opioid**	Yes	23	125	0.776 (.482,1.40)	0.405	0.311(0.112,0.868)	0.026
	No	28	196	1		1	
**Non-opioid**	Yes	24	119	0.663(0.366,1.20)	.175	0.284(0.102,0.790)	0.016
	No	27	202	1		1	
**Antifungal**	Yes	14	45	2.262(1.136,4.509)	0.020	1.487(0.662,3.341)	0.337
	No	37	276	1		1	

*CI* Confidence Interval, *COR* Crude Odds Ratio, *AOR* Adjusted Odds Ratio * = *p* value *<* 0.05*statistically significant with IPT utilization *p* ≤ 0.05

## Discussion

Isoniazid preventive therapy has been one of the four main strategies of TB prevention in both HIV positive and negative patients since the introduction of Isoniazid [15]. The overall prevalence of IPT utilization among HIV positive clients used ART medication was found to be (62.1%). This finding was lower than from a retrospective study conducted in Arbaminch Hospital (68%) [13]. However, our finding was higher compared with a cross-sectional descriptive study studies conducted in Tigrai 20% [16], a qualitative study was done in AddisAbaba 30%. Purposive sampling technique was carried out to select the participants based on exposure to their patients in all circumstances [17, 18]. In addition, Addis Ababa among 14 public health facilities A. A (77%) [19], Prospective cohort done in Cambodia (78%) [20],and a cross-sectional study conducted in Swaziland (89.4%) [21]. However, higher than studies done in south Ethiopia (50.9%) and South Africa (69%) [13, 22]. This difference might be due to the lack of reinforcement by local health officials and stakeholders working in TB was affecting the implementation of the IPT policy. The success of IPT implementation is mainly dependent on continuous capacity building and monitoring. Health providers perceived that insufficient attention is being given to IPT by responsible bodies. In some hospitals sensitization workshops, technical training, and operational guidelines were not provided [16, 23]. Moreover, the study design and sampling techniques of the studies might be contributed to the difference. Therefore, to increase the IPT implementation and scale-up program clients’ awareness should be increased about the adherence through counseling and increased knowledge patient’s awareness of the importance of INH and TB and HIV association and its severity.

In this study, among the IPT utilized participants, 4.8%of them were developed tuberculosis. This finding was similar to a study done in Jimma University medical center, Ethiopia (3.78%) [14]. However, this study was higher compared with retrospective cohort studies conducted in Ethiopia Jimma, (2.7%) [24], and Zimbabwe (0%) [25].

In our study, the proportion of TB developed individuals among HIV positive clients who took and completed IPT for 6 months was 2 (15.40%). This finding was higher than from the study done at Arbaminch hospital (8.7%) whereas from those who didn’t taken IPT 5(9.80%) study participants had acquired TB infection which was lower than the study done at Arbaminch hospital (27.80%) those difference may be due to sampling size difference, awareness, and information [13]. However, this indicates that the completion of IPT in HIV infected clients significantly reduced TB incidence by 69.2% when compared to those who were developed TB before completing their IPT 11(84.6%). Moreover, IPT is one of the key interventions recommended by WHO in 1998 to reduce the burden of TB in PLHIV; yet implementation of IPT had been very low in different countries [26].

In this study, 18(4.80%) study participants had developed TB after initiated of ART that was higher than the study done among HIV-infected patients receiving medical care at 29 public clinics in Rio de Janeiro, Brazil (1.4%) [27]. This difference may be due to sampling size differences, knowledge and awareness. Lack of training on INH and no one showing responsibility for monitoring the program are the commonly mentioned reasons for low prescription rate. Care providers opinion for factors affecting adherence and utilization of IPT.

## Conclusion

The utilization of INH among people living with HIV in Fresemaetat primary hospitals was low. Lack of appointment per demand, lack of training, busy clinic and non-sustainabledrug supply and lack of trust when the supply is inconsistent after initiation for few months were the possible reasons for the low utilization rate of IPT. Therefore, Increase provider‟s knowledge on IPT through training and monitoring and evaluation,consistent IPT delivery system and introducing new tools into practice in line with the global plan to stop TB is important.

## Data Availability

The datasets used and analyzed during the current study are available from the corresponding author on reasonable request.

## Abbreviations

ART: Antiretro Viral Therapy
BMI: Body Mass Index
CPT: Co-Trimexazol Prophylaxis Therapy
CSA: Central Statistical Agency
HIV: Human Immune Virus
IPT: Isoniazid Prophylaxis Therapy
SPSS: Statistical Package for Social Science
TB: Tuberculosis
WHO: World Health Organization
